# Mental health among adolescents exposed to social inequality in Latin America and the Caribbean: a scoping review

**DOI:** 10.3389/fpubh.2024.1342361

**Published:** 2024-04-10

**Authors:** Johanna Carolina Sánchez-Castro, Laura Pilz González, Saidy Eliana Arias-Murcia, Viviana Andrea Mahecha-Bermeo, Christiane Stock, Katherina Heinrichs

**Affiliations:** ^1^Institute of Health and Nursing Science, Charité – Universitätsmedizin Berlin, Corporate Member of Freie Universität Berlin and Humboldt-Universität zu Berlin, Berlin, Germany; ^2^Faculty of Nursing, Universidad El Bosque, Bogotá, Colombia; ^3^Faculty of Humanities, Social Science and Education, Otto-von-Guericke-Universität Magdeburg, Magdeburg, Germany

**Keywords:** adolescents, mental health, psychological phenomena, social discrimination, social inequality, social sciences, social vulnerability, well-being

## Abstract

**Background and objective:**

Adolescents from Latin America and the Caribbean grow up in a context of social inequality, which diminishes their well-being and leads to impaired emotional-cognitive development. To understand the problem, it is important to synthesize the available research about it. This study aims to explore the knowledge about adolescents’ mental health in Latin America and the Caribbean exposed to social inequality.

**Methods:**

A systematic scoping review was conducted encompassing a search in five databases (Medline, CINAHL, PsycINFO, Scopus, and LILACS) in June 2022. Articles of various typologies were included without time limit. After two rounds of screening, relevant data were manually extracted and synthesized into self-constructed themes using thematic analysis.

**Results:**

Out of 8,825 retrieved records, 42 papers were included in the final review, with a predominance of quantitative approaches. The synthesis revealed two main analytical themes: (a) defining social inequality, wherein intersecting inequalities produce discrimination and determine conditions for social vulnerability; (b) social inequality and mental health, which highlights the association between socio-structural difficulties and emotional problems, amplifying vulnerability to mental ill health and poor mental health care.

**Conclusion:**

The scientific evidence reveals that social inequality is related to impaired well-being and mental ill health on the one hand and a lack of access to mental health care on the other hand.

## 1 Introduction

Adolescents have different roles in society: on the one hand, they represent the development of countries as they will be the adults of tomorrow, so they are the workforce of the future and the caregivers of generations to come (1, 2). On the other hand, adolescence is portrayed as one of the most stressful phases in life (3, 4) because it is connected to many challenges, such as hormonal and physical changes (5, 6). In addition, the desire for independence, the establishment of an own identity, and/or the development of autonomy are also characteristics of this phase (6, 7).

In the construction of their different roles, adolescents are particularly sensitive to the social, economic, and cultural environment in which they grow up (8). Scientific evidence shows that the socioeconomic background of their countries affects adolescents’ life satisfaction and quality of life (9). As a result of an environment marked by poverty and discrimination, youths experience insecurity and hopelessness as well as the feeling of not having the necessary resources to fulfill their life projects (4, 10, 11). This social vulnerability, combined with the chaotic phase of adolescence, creates mental stress scenarios, and in this process, serious mental health problems may emerge (10, 12).

There is extensive evidence in the literature about how different aspects of social inequality are linked to mental health problems in adolescents. Studies conducted in Germany and Australia demonstrate that children and adolescents with low socioeconomic status (SES) and family difficulties have a higher risk of mental health problems (13, 14). Another study shows that adolescents in South Africa living in poverty face stressful situations that often lead to symptoms of depression and anxiety (15). In research involving Chinese adolescents, it was found that those with advantageous socioeconomic conditions such as social support and adequate financial resources show a better mental health status than less privileged adolescents (16).

In Latin America and the Caribbean, adolescents grow up in a context of social inequality, which affects people’s lives in different ways. One type of social inequality is economic inequality, which includes income and property (17, 18). Other forms of structural inequality hinder the fulfillment of human rights, the development of skills, educational and job-related opportunities, and autonomy (17, 18). Contexts marked by structural inequality – characterized by low income, unemployment, social exclusion as well as poor access to health services, education, and housing – undermine the quality of life and lead to discontent among the most vulnerable populations (17, 19). Especially among young people, inequality diminishes the possibilities to participate in society and to manage the transition to adulthood successfully, i.e., reaching their full potential and ultimately contributing to the improvement of society (1, 20). Thereby, social inequality allows for the violation of adolescents’ rights, especially in families with low SES (21).

So far, research on the association between social inequality and adolescents’ mental health has primarily focused on high-income countries (22, 23). Despite Latin America and the Caribbean being recognized as the region with the highest levels of inequality worldwide (24, 25), only one literature review has examined the association between socioeconomic inequality and mental disorders, covering the general population, including adults (26). This highlights a clear gap in the synthesis of the relationship between social inequality and mental health in adolescents from Latin America and the Caribbean.

Therefore, the present study aims to explore the available knowledge about the mental health of adolescents exposed to social inequality in Latin America and the Caribbean. It also aims at identifying the definitions of social inequality used in literature available on the topic. As a guide for reporting the results of this study, we used PRISMA Extension for Scoping Reviews (PRISMA-ScR) (27).

## 2 Methods

Due to the complexity and heterogeneity of the research topic and to account for the conceptual aim of this research, it was considered optimal to conduct a scoping review (28). We followed the six-stage scoping review framework proposed by the Joanna Briggs Institute (JBI) (28), which consists of the following stages: (1) identifying the research questions; (2) identifying potentially relevant studies; (3) study selection; (4) data extraction; (5) analysis of evidence; (6) presentation of the results. The detailed protocol of this study is available online (29).

### 2.1 Search strategy

The search structure was designed based on the PEO strategy (Population, Exposure, Outcome) (30). The population of interest is defined as adolescents born and living in Latin America and the Caribbean. Social inequality, serving as exposure, is defined as social difference in living conditions and essential services among different social groups, encompasses social exclusion, discrimination, power imbalances, migration or forced displacement, poverty, and violence (31). The outcome is the adolescents’ mental health, defined as the individual’s sense of well-being, self-efficacy, autonomy, and competence, along with the development of social relationships (32, 33).

The databases and keywords were determined with the support of an expert in Information Literacy Education/Systematic Reviews. We carried out searches in Medline (via OVID), CINAHL, PsycINFO, Scopus, and LILACS for articles resulting from primary research studies, systematic reviews, and reflection articles, including expert opinions on the subject in English, Spanish, and Portuguese. We did not limit our search by publication date. The search strings were adapted for the respective databases, but remained comparable. As an example, the full electronic search strategy for Medline is available in Appendix 1.

The following criteria were applied in selecting the literature included: 1. The aim of the study refers to adolescence, which extends from 10 to 19 years of age (34). However, we include samples exceeding this range as well as adult samples as long as the purpose of the study clearly relates to the situation of adolescents. 2. The study focuses on a population born and living in Latin America or the Caribbean. 3. The study explains or analyses the association of social inequality with adolescents’ mental health.

### 2.2 Study selection

All databases were searched on 30 June 2022. Afterwards, all records were imported into Endnote X9.3.3 (Clarivate Analytics) to find and eliminate duplicates. Subsequently, all remaining literature was uploaded to the online systematic review platform Rayyan Systems, Inc. for further screening, first title and abstract, then full-text. We employed a team approach in this stage: due to linguistic competence, two of the researchers screened the results in English and Spanish (JCSC and LPG) and two in Portuguese (SEAM and VAMB). Discrepancies between the reviewers were reconciled through direct discussion at each phase until consensus was achieved.

Further sources were identified through a backward literature search by reviewing the references cited in the articles included (28). The main researcher (JCSC) checked the additional titles for inclusion criteria, and those eligible were included in the data extraction and analysis phase. A team approach was not used for the screening of these articles as they came from papers already included in the review, which represented an indirect screening. In addition, previous discussions provided experience for single-author decision making at this stage.

### 2.3 Data extraction and analysis

Information from the included records was extracted into a charting table in Microsoft Excel by JCSC. The following data were extracted: author, year, country, type of article, aim, design, study population, method/data, assessed variables, limitations/bias, and key findings regarding the conceptualization of social inequality, mental health approach, and associations between social inequality and mental health in adolescents in Latin America and the Caribbean. Subsequently, the information in the charting table was summarized alongside the general information of the studies.

For the qualitative synthesis, a process of inductive thematic construction for scoping reviews was used, consisting of manually identifying and classifying relevant and recurring patterns in the extracted text segments. These patterns were then reorganized into themes, with descriptive sub-themes when necessary, by which we answer our research questions (35).

## 3 Results

### 3.1 Scope, nature, and distribution of literature

**Figure 1:**
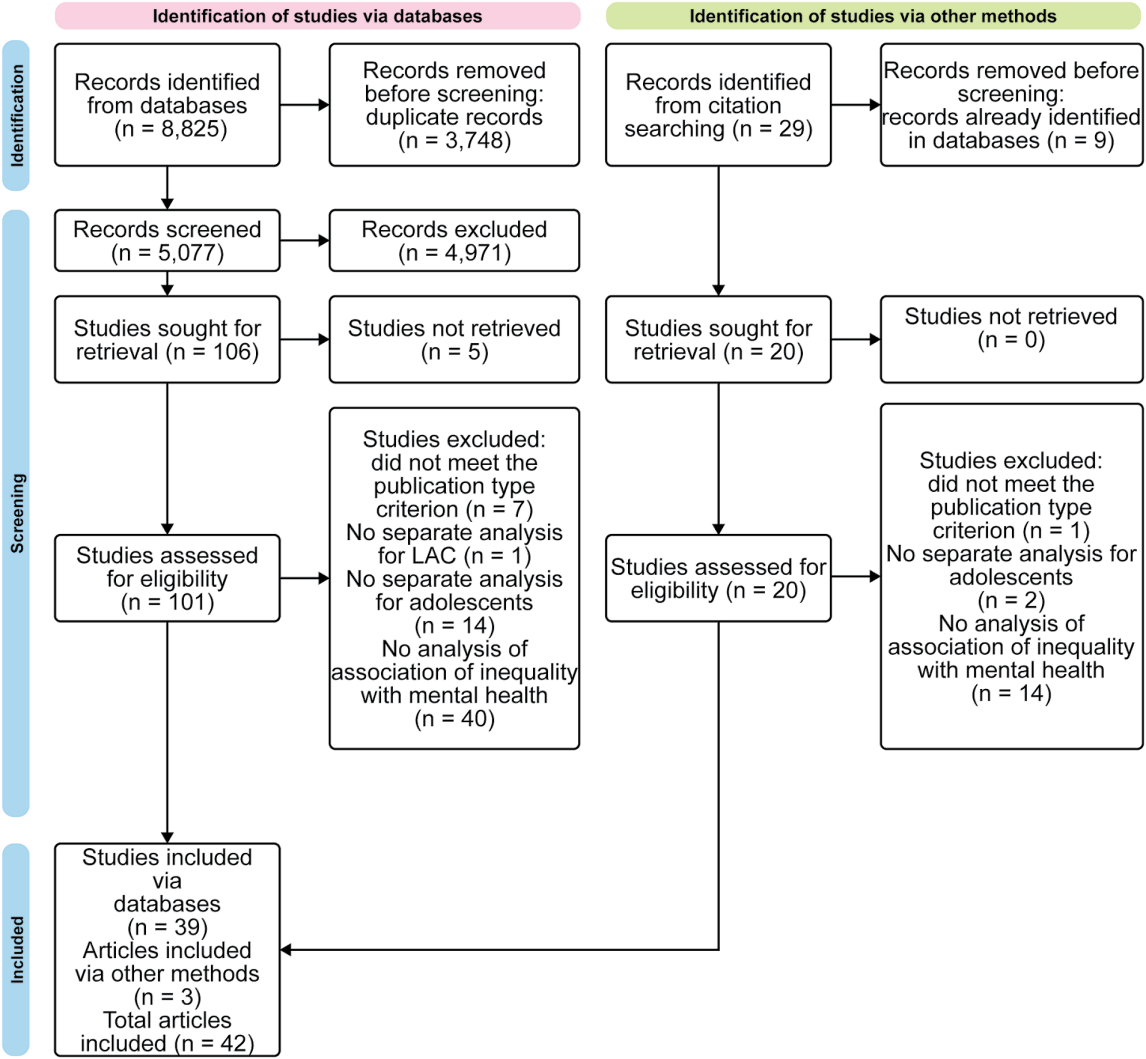
Flow diagram of selection process (designed on miro.com).

Figure 1 depicts the selection process (27, 36). The initial database search yielded 8,825 articles. After elimination of duplicates, 5,077 studies remained. The title and abstract screening resulted in 106 articles. A total of 101 articles was retrieved as full text, the respective screening resulted in the final count of 39 articles. Another three publications were added by manually scanning the references of the included publications. In the end, a total of 42 articles met the inclusion criteria.

Supplementary Table S2 provides the characteristics of the 42 articles included. The articles were published between 1989 and 2022. When classifying the year of publication by decades, a greater number of publications was found in the period from 2011 to 2020 (*n* = 23). The majority of studies were conducted in Brazil (*n* = 19), with the remaining articles written by authors in Colombia (*n* = 5), Mexico (*n* = 5), Chile (*n* = 4), Ecuador (*n* = 4), Nicaragua (*n* = 2), Peru (*n* = 2), and one study included two countries, Colombia and Mexico. The predominant language was English (*n* = 28), followed by Spanish (*n* = 8) and Portuguese (*n* = 6). We identified 39 original articles, one letter to the editor, one description of the implementation of a health care project, and one review of the scientific literature. Among the original articles, most used a quantitative design (*n* = 29), 27 were cross-sectional studies, one cohort study, and one panel study. A total of eight studies were conducted qualitatively, and two followed a mixed methods design.

### 3.2 Thematic findings

As a result of the thematic analysis, two main themes emerged: 1. Defining social inequality; 2. Social inequality and mental health. For the second theme, the construction of sub-themes “social inequality threatening well-being,” “social inequality and mental ill health,” and “mental health care in social inequality environments” was significant to deepen its description.

#### 3.2.1 Theme 1: Defining social inequality

**Figure 2:**
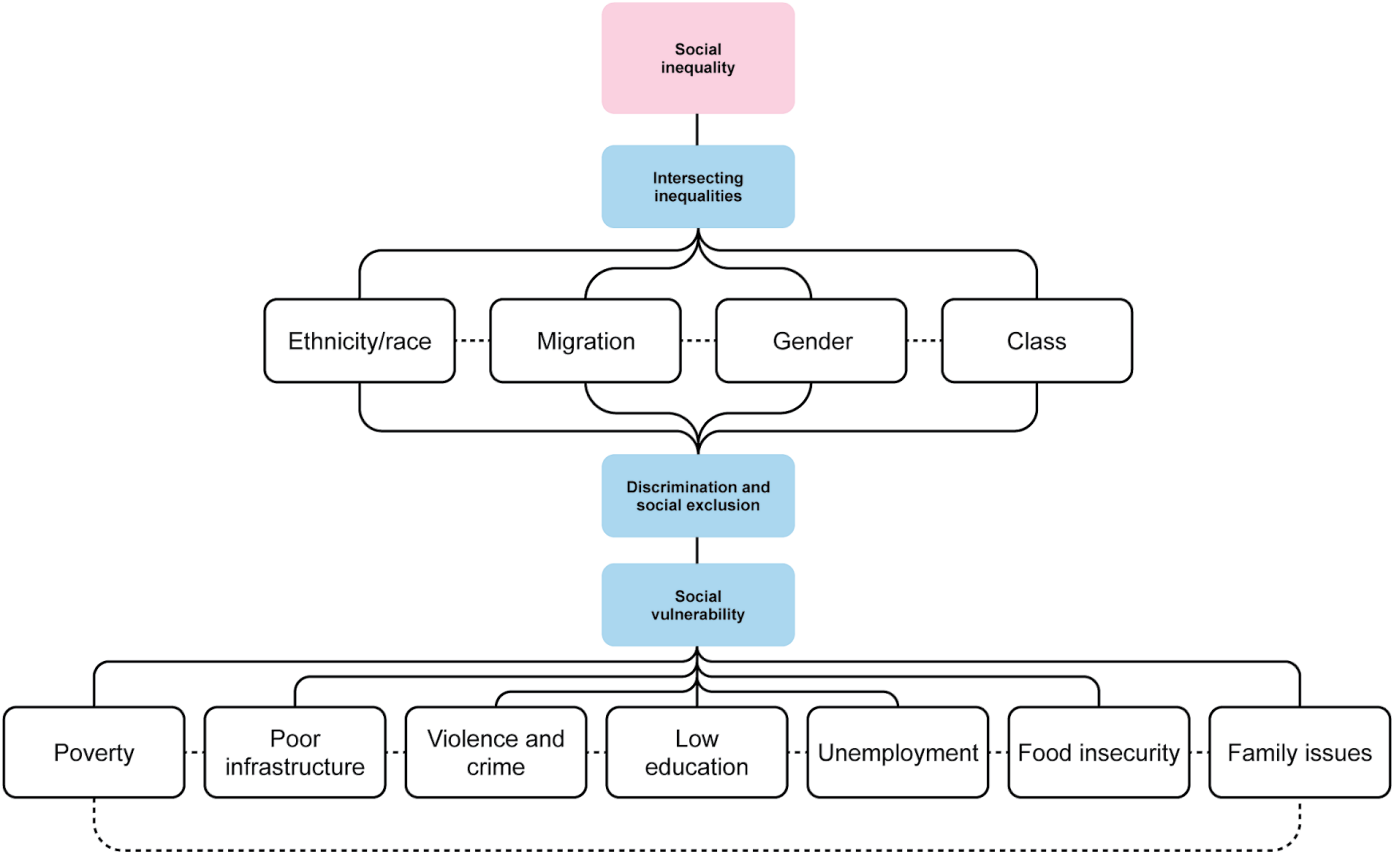
Theme 1: Defining social inequality (designed on miro.com).

Across the 42 studies included, we identified varied approaches in defining social inequality. The concepts mapped in this theme are presented in Figure 2. Social inequality seems to emerge from the interplay of ethnicity/race, migration, gender, and class. These are social categories that play a decisive role in shaping the social environment.

Regarding ethnicity/race, it is described how populations with ethnic-racial background often experienced inequitable treatment (37, 38). One of the articles reports people being mostly white in the central areas of the cities, where socioeconomic conditions were favorable (better parental education and formal employment) (39). However, in populations of African descendants, lower levels of structured education and low income were prominent (40).

Another relevant phenomenon was migration due to various reasons, e.g., forced migration related to conflict and war, rural–urban migration, international migration that might be south–south among Latin American and Caribbean countries (between countries or regions located in the global South, involves people moving from one developing country to another) or south–north (from less economically developed regions to more economically developed countries) (41–45). Migrant population usually concentrated in the peripheral neighborhoods embodied by social vulnerability (42, 45).

Few studies investigated the role of gender in perpetuating social inequality. This research sheds light on traditional patriarchal norms (sometimes based on religious belief), exposing women to greater social and financial vulnerability (46, 47). When gender inequalities were conjoined with socioeconomic stratification (class), compelling evidence of social inequality emerged. For instance, among groups of economically disadvantaged adolescents who were obliged to work, female adolescents were usually engaged in unpaid activities such as stay-at-home care for younger siblings and household chores, while male adolescents were engaged in paid work (46, 47).

In several studies, the concept of SES was used to classify the participants according to their class, for example, based on goods and assets of the family, and/or schooling of the head of the household (37, 45, 48–56). The type of school such as municipal or subsidized school and paid school was also used to define the SES (57). In these papers, this classification was based on the location of the schools or households like low-, middle-, or high-income areas (58, 59).

In social environments where ethnicity/race, migration, gender, and/or class intersecting inequalities emerge, the affected population became marginalized, facing exclusion from state provisions and broader societal engagement (50, 60, 61). Notably, impoverished adolescents were distinctively affected by discrimination, social exclusion, and impediments in the realization of fundamental human rights (37, 61–65).

Furthermore, individuals facing intersecting inequalities were susceptible to social vulnerability, which manifests through challenging life circumstances (20, 40, 46, 60, 62, 64–70). Several articles highlight poverty as one of the most significant determinants of social vulnerability. Poverty has been widely associated with income shortfall. More recently, this concept has been expanded to the consumption of goods and services (71). However, the essential definition of poverty recognizes it as a fundamental life condition that affects social, economic, and environmental relations and determines the degree of social inequality (20, 39, 40, 47, 54, 57–59, 61, 62, 65, 68, 71–74).

Moreover, a number of studies have reported that poor infrastructure is characteristic for social vulnerability. Two studies point out that neighborhoods dominated by low social status presented hazardous life circumstances which included residences in a precarious state of repair and with insufficient rooms and thus hindering privacy, lack of piped water, sewage, and electricity as well as inadequate garbage collection (68, 75). Poor infrastructure is also related to lack of public transportation, besides leisure, education and health centers (39, 47, 58). Furthermore, in 11 articles, disadvantaged neighborhoods were characterized by high levels of insecurity (violence and accidents), drug trafficking, urban crime, police raids, assaults, robberies, and shootouts (20, 39, 42, 58, 59, 62, 63, 65, 67, 70, 76).

Social vulnerability was further manifested in the disparities in educational attainment among individuals from low, middle, and higher socioeconomic classes (39). The access to education among poor adolescents is erratic, reflected in their low school enrolment (20, 42, 46, 62, 65, 70, 71). This phenomenon also occurs in the parental (household head) generation among disadvantaged groups (40, 58, 66).

Similarly, numerous studies reported that caregivers’ loss of employment or engagement in low-status occupations, particularly within informal, illegal, or low-paying sectors, contributed to an increase in the family’s social vulnerability (20, 39, 40, 42, 58, 66, 70, 72). Furthermore, the need of increasing family income could push young individuals into premature employment, which could be harmful because it interferes with their education and personal development (46, 65, 76). Unemployment and economic risk lead to food insecurity and insufficiency and thus, to adolescents suffering from hunger (20, 43, 58).

Social vulnerability is also reflected in family issues. Three author teams identified family disruption as an indicator of socioeconomic disadvantage (40, 47, 67). Across 11 articles, social vulnerability within the family setting is described through various factors, including parental conflict and divorce, the presence of a stepfather, extended family or single-parent family, or mother-only households (39, 40, 42, 47, 58, 66). Two-parent families were expected to provide much more stability, with changes in the family structure considered as a risk factor (39, 58). Fragile families are also characterized by domestic violence and familial addiction (62, 63). In Latin America and the Caribbean, the breakdown of the family structure is common due to international migration, when adults leave their country of origin in search of employment and resources, leaving their children behind in the care of grandparents or other relatives (41, 44).

#### 3.2.2 Theme 2: Social inequality and mental health

**Figure 3:**
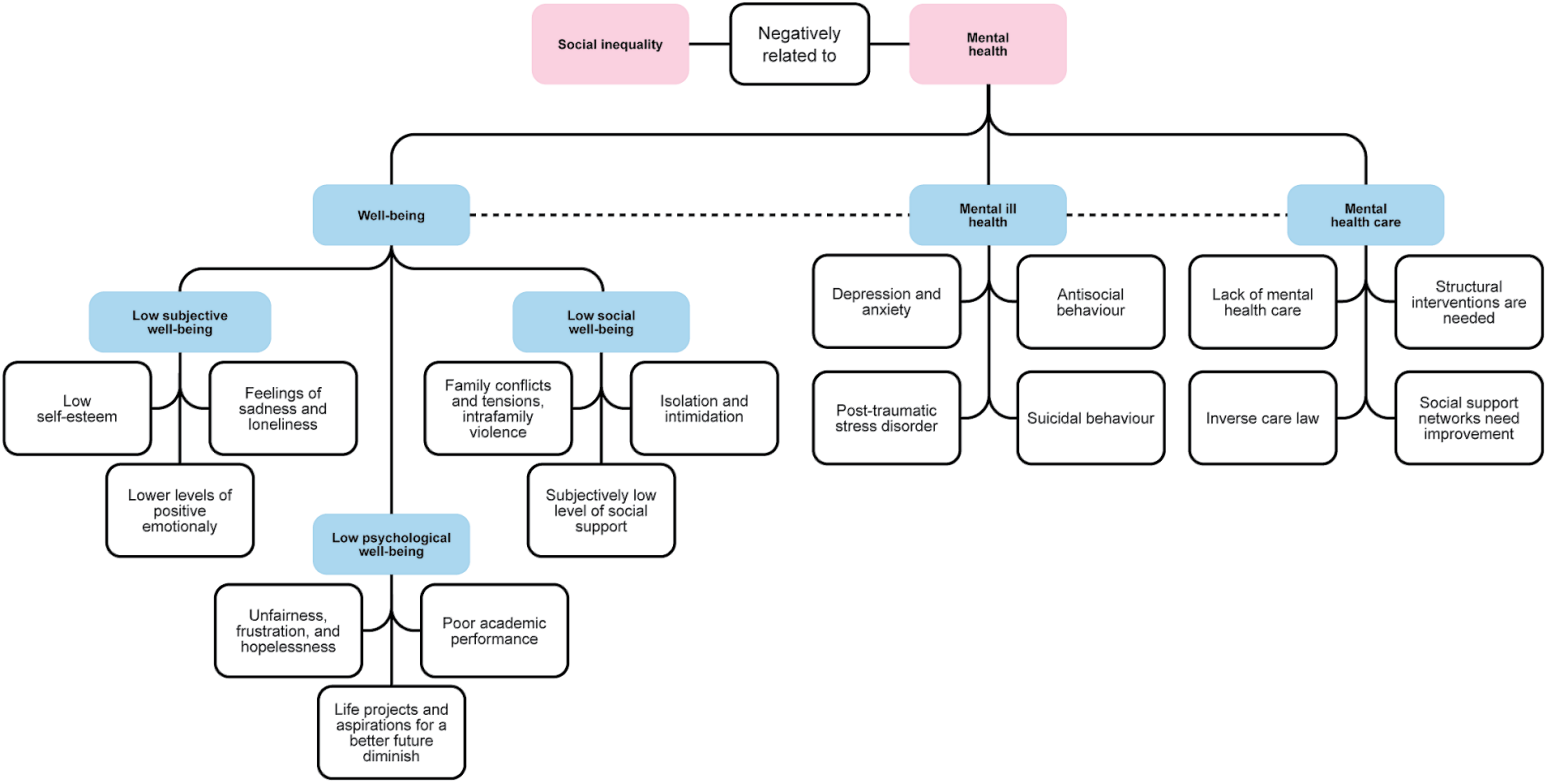
Theme 2: Social inequality and mental health (designed on miro.com).

Across various studies, there was evidence that social inequality was negatively related to adolescents’ mental health. The concepts mapped in this theme are presented in Figure 3. Sub-themes were 1. Social inequality threatening well-being: how social inequality is related to the positive mental health approach, emphasizing concepts like well-being and quality of life. 2. Social inequality and mental ill health: focusing on a clinical standpoint, analyzing symptoms and/or mental health disorders that are prevalent among adolescents experiencing social inequalities. 3. Mental health care in social inequality environments: the lack of access to mental health care for adolescents facing social inequality.

##### 3.2.2.1 Social inequality threatening well-being

A number of studies reported that well-being was a key component of mental health and was based on the degree that each individual assesses their life circumstances, depending on the possibility to cope with and assimilate life events (49, 59, 65). There are three types of well-being: subjective well-being, psychological or eudaemonic well-being, and social well-being (70). Here, we map the diverse ways in which social inequality poses challenges to overall well-being, encompassing the intricate interplay between the abovementioned types.

Some evidence showed that the higher the social inequality, the lower the well-being (49, 59, 65, 71, 73). We found research on how intersecting inequality relates to mental health. Two studies described how discrimination based on ethnicity/race generates negative emotional responses, thereby raising difficulties for the proper development of mental health (37, 38). In particular, two papers reported how adolescents who were left behind after their parents’ migration reported feelings of sadness, loneliness, and behavioral problems (41, 44). Moreover, patriarchal norms affected the communication and expression of feelings among female adolescents, especially in relation to sexuality, creating feelings of inferiority in young women (47). In this regard, a synergy could be seen between gender and SES, with women from schools in poorer social conditions having less self-esteem and stronger feelings of anxiety and sadness (57). In addition, adolescents’ quality of life was reduced by high levels of emotional stress resulting from the lack of social and economic resources, representing early adversity for youths (42, 52, 53, 55, 56, 59, 65, 71, 74). The result was social vulnerability characterized by stressful events and lower levels of positive emotionality (40, 53, 58, 74). Social vulnerability was associated with aggressive behavior (20, 65, 67) with the affected adolescents becoming victims or aggressors, perpetuating the vicious circle of violence (59).

Furthermore, increased poverty and perceived social exclusion were associated with lower self-esteem, perception of having less social support, and feelings of isolation and intimidation (37, 38, 58, 65) as well as feelings of unfairness, frustration, disrespect, anger, and hopelessness (42, 59, 69). In general, lack of opportunities and sometimes also of information hindered adolescents to follow their life projects and aspirations for a better future (better conditions than those of their caregivers) (65, 68, 71).

Besides, well-being was constantly threatened in hostile environments characterized by high levels of insecurity, violence, and crime as well as precarious infrastructure such as substandard housing, inadequate sanitation, and low quality of local services (48, 65, 67, 72, 77). Having computer or internet access was essential to perform academic activities, but not available to disadvantaged adolescents, leading to daily stress (50).

Socially vulnerable families were more likely characterized by family conflicts and tensions, intra-familiar violence, illnesses, single-parent families, large families, and unemployment, which were considered as stressful situations impairing adolescents’ mental health and quality of life (50, 54, 56, 63, 67). Likewise, low levels of parental education and little or no paid work might impose stress and reduce the mental stability of adolescents (49, 65, 77).

These life circumstances played a central role in adolescents’ academic performance as well: in schools where students had better socioeconomic conditions, their cognitive and psychosocial skills were better developed than in schools located in poor areas, generally public schools (60, 65). Although one study reported that educational work with adolescents could help them improve their self-esteem as they felt valuable to their family and community (76), this was different when measured in relation with other work circumstances. For instance, adolescents who could not study because they have to work or those not in education, employment, or training (NEET) are affected by social vulnerability that puts their mental health at risk since they are unable to meet social expectations, and this generates feelings of dissatisfaction with life (66). In addition, when economic difficulties force the youngest members of the family to work, they suffer from the responsibility to provide for a family far too early (46).

The situation of street adolescents was complicated in its own way, since most of them fled from the negative situation at home, thinking that in the street they would find autonomy and freedom to fulfill their life expectations (61). However, they ended up facing the sense of having lost family protection, resulting in feelings of shame and loss, exclusion, discrimination, and violence (61). In addition, they tended to develop inappropriate behaviors such as taciturnity, hostility, and mistrust (63).

##### 3.2.2.2 Social inequality and mental Ill health

Mental health was often investigated from a psychopathological perspective. The common terms used were “mental ill health,” “common mental disorders” or “psychiatric disorders” in the literature in English and its equivalent translation in the other two languages (39, 42, 52, 64, 69, 71). Here, we present different associations found between social inequality and mental health problems.

Several studies reported that socioeconomic adversities were associated with mental ill health (40, 42, 69, 74, 75). Less formal education and parental unemployment were found as strong predictors of high rates of mental disorders (40, 50, 71, 72, 74). While one group of researchers identified that a significant portion of depressed adolescents were associated with functional families (54), in most studies, disruptions in the family structure played an important role in the vulnerability of internalizing and externalizing problems (40, 43, 50, 71, 72). Although in one of the studies, no significant difference between inner city and outer city areas was found (39), living in structurally deprived neighborhoods or in under-served rural areas was shown to be associated with psychosocial deprivation and thus, with the vulnerability to develop mental illness in two other papers (72, 74). Research in adolescents identified as NEET showed that they have greater odds of mental ill health in connection with disengagement from social, educational and working life (66).

Poverty and social vulnerability were associated with depressive and anxiety symptoms (20, 40, 51, 64). This was the case in the children of ragpickers who reported how the constant presence of garbage in their homes caused a feeling of constant discomfort with their environment (72). Furthermore, young people who experienced racial discrimination showed higher rates of depression compared to those who never felt discriminated (37). Working adolescents showed higher levels of anxiety or depressive symptoms than those who did not (46). Food insecurity and hunger were also associated with depressive symptoms in adolescents (43).

Further important mental health issues among poor adolescents were social problems, aggressive behavior, and antisocial behavior (ASB) (20, 40, 51, 67). In most cases, male adolescents were more likely to externalize their problems (39). Family conflict, in particular when parents used physical punishment as disciplinary strategies, confers risk for aggressive behavior and other externalizing disorders (40, 67). In addition to the psychiatric diagnoses mentioned above, it is important to remark the high probability of developing post-traumatic stress disorder (PTSD) in street adolescents who experienced extreme inequality and thus, vulnerability (62).

Some studies reported how the effects of social vulnerability described above can also trigger suicidal behavior (47, 56, 58, 72). Being a child of a ragpicker, racial discrimination, hunger, and low parental/guardian involvement are risk factors for self-injury (37, 43, 72). In addition, the absence of social support networks can be a risk factor for suicidal behavior (56, 66). One research on the socioeconomic and political determinants of suicide in adolescent females showed an association of turbulent political situations, traditional patriarchal norms, and the weakening of religions with suicidal behavior (47).

##### 3.2.2.3 Mental health care In social inequality environments

Few studies investigated how families, health system, society and governments recognize and attend to adolescents’ mental health. Two studies reported that sometimes mental health issues were denied, e.g., by parents who reported that their children’s complaints were somatic in nature (20, 75). Furthermore, mental health care was shown to often be lacking in poor areas (40, 57, 65, 71). Likewise, lower income populations with higher morbidity received less than the minimum amount of mental health care, while privileged populations received excessive assistance – this is known as “inverse care law” (40, 48, 56, 57).

Finally, it was reported that improving adolescents’ mental health required major efforts at different levels. First of all, structural and systematic interventions were needed to address inequality and social vulnerability (44, 52). At the same level, it was necessary to promote dignified and well-paid employment for caregivers as well as strategies to increase family cohesion (40, 44, 65, 67). The development of social support networks in which adolescents and their families can trust was highly recommended (42, 45, 65, 70). For instance, the construction of youth organizations that motivate them to optimize the utilization of leisure time and achieve social inclusion removing discrimination of all kinds (37, 42, 44, 45, 70).

## 4 Discussion

In this scoping review, we described the concept of social inequality based on intersecting inequalities and social vulnerability of adolescents as well as the resulting life circumstances in Latin America and the Caribbean. Furthermore, this scoping review shows that social inequality in different countries is associated with negative emotions related to the low well-being of adolescents, harms their self-esteem, hampers the life projects viability, and fosters feelings of isolation. It is also revealed that these contexts are related to the presence of mental illness and suicidal behavior. In addition, we highlight the lack of access to health promotion, prevention, and care services in mental health for young people experiencing social vulnerability.

### 4.1 Defining social inequality

Through the thematic analysis of the articles included in this review, we examined the broad and complex concept of social inequality, revealing how important it is for Latin American and Caribbean societies, since this is considered to be a region with highest rates of inequalities compared with other areas in the world (24, 25, 67). The unequal conditions in which people in the region live have caused social injustice and difficulties for the population to have opportunities maintaining their quality of life and achieving individual and collective well-being (78).

To understand the complexity of the concept of social inequality, we consider the approach of intersectionality, which has been used in previous research to analyze social inequality in Latin America, for example by Baquero Melo (79). This analytical framework acknowledges the diversity inherent in human experiences by contemplating the overlapped dimensions of ethnicity/race, migration, gender, and class (79). In this approach, it is recognized that these categories are inseparable (31). Previous studies have reported that adolescents in Latin America growing up in a context that presents them obstacles to different levels, resulting in the accumulation and reinforcement of inequality (79, 80).

Social inequality is also closely linked to discrimination, with certain populations experiencing unfair and unfavorable treatment based on characteristics such as low SES, ethnicity/race, migrant background, or gender identity (Erin (81, 82)). In Latin America and the Caribbean, research indicates that disadvantaged communities encounter limited access to opportunities and resources due to the prejudices and stigmatization present in society. This means that social inequality and discrimination creates a self-reinforcing cycle that hinders the comprehensive human development of these marginalized populations (17, 80, 81). In this way, a profound acknowledgment arises concerning the intensified infringement upon the fundamental rights of certain groups (25, 83).

We raise a warning regarding unfavorable life circumstances specifically concerning poverty, poor infrastructure, violence and crime, limited educational opportunities, unemployment, food insecurity, and family issues. These factors have been found to have detrimental associations with adolescents’ mental health (84). Furthermore, given the lack of evidence in rural areas in this scoping review, future primary studies should focus on examining social inequality in these regions. It is essential to consider the challenges posed by limited access to healthcare, education, and other vital services in rural territories. By addressing these issues, we can gain valuable insights into social disparities and develop targeted interventions to tackle these challenges effectively. This realization poses a significant challenge in developing effective public policies and attention programs addressing the needs of the most vulnerable populations. The aim is to dismantle the underlying structures of social inequality and thus, to ensure equitable access to human rights and better living conditions for all (17).

It is acknowledged that consolidating all countries in the region into a single study of social inequality may oversimplify matters, considering the distinct historical, social, political, and cultural processes unique to each nation. However, gaining a general overview of the situation remains valuable, as it aids in understanding the regional profile and furnishes the essential knowledge needed for formulating comprehensive public policies. Moreover, it presents an opportunity to advance research by exploring the specific characteristics of each country in future studies.

### 4.2 Social inequality and mental health

The associations outlined herein are contextualized within Latin America and the Caribbean, where historical processes of colonization contribute to racial hierarchies, discrimination, and the marginalization of diverse ethnic groups (85). Furthermore, traditional gender roles prevail, with men often being the authoritarian heads of households, while women are relegated to secondary positions (86). Additionally, economic and political shifts, frequently accompanied by violence and criminality, have shaped the socio-economic situation of whole peoples and, in many cases, have driven migration both within and beyond national borders (87). It is therefore essential to comprehend mental health as an experience influenced by all the social phenomena inherent to the region. From this perspective, we can identify processes aimed at enhancing the living conditions and health of adolescents residing in the area (88).

In this scoping review, three distinctive sub-themes regarding the association between social inequality and mental health have emerged, delineated based on the various aspects examined in the included studies. The first sub-theme is based on the well-being and positive mental health approach, studies from this perspective refer to mental health in terms of quality of life, life satisfaction, and social relationships, explaining that mental health goes beyond diagnosing illnesses and requires attention based on health promotion and psychosocial work (89).

Well-being appeared as the prevailing concept frequently employed to define positive mental health within the scope of the included articles. Moreover, we found diverse well-being dimensions that contribute to a more nuanced understanding of the relationship between social inequality and mental health. First, subjective well-being was most commonly used by researchers and has two essential components: (a) the affective or emotional component which includes a balance between positive and negative affectivity or emotional responses and (b) the cognitive component characterized by the evaluation of global satisfaction with life (48, 57, 59, 73, 76). This scoping review highlights a consistent pattern indicating that adolescents residing in contexts characterized by social inequality present diminished levels of subjective well-being. To assess this phenomenon various instruments were used, encompassing evaluations of quality of life, self-esteem, and life satisfaction. The evidence indicates that social vulnerability serves as a potent stressor, fundamentally linked to the experience of reduced positive emotionality and self-esteem among adolescents not only in Latin America and the Caribbean but other social inequality contexts around the world (4, 90).

The second dimension is psychological or eudaemonic well-being. Adolescents facing conditions of poverty, poor infrastructure, limited educational opportunities, and unemployment confront significant challenges in fostering hope for the future and realizing their personal aspirations (68, 70). This is comparable to adolescents from other regions in the world. For example, a study among adolescents from families with financial difficulties in China found that adolescents’ future orientation is under threat (91). The results of this scoping review in a Latin American context supported it. In the included literature there is evidence on how the scarcity of socioeconomic resources and the adversities stemming from the intersecting inequalities of ethnicity/race, migration, gender, and class can impede the adolescents’ autonomy and freedom of choice concerning life goals or finding their own way for investing an effort (61, 70).

Thirdly, social well-being refers to the capacity to be part of society and to build social networks (70). The institutions of family, community, school, the state, informal support networks, and the relationships formed between their members are a key component of well-being and the fulfillment of human needs (61, 68). For adolescents, family is one of the most important protective factors for mental health through the dimensions of cohesion, affectivity, hierarchies or roles, communication, decision making, and conflict resolution (40, 43, 54). Family marks an important influence on the development of the individual’s personality (40, 44, 75). Consequently, households with adolescents experiencing family disintegration, domestic violence, economic hardships, and insufficient resources to fulfill fundamental nutritional and housing requirements pose a significant concern, not only for social well-being but also due to their connection with other dimensions of well-being. These adversities are closely linked to diminished levels of self-esteem, self-efficacy, and self-concept (92).

It is imperative to underscore the profound challenges faced by populations affected by intersectional discrimination. According to numerous research studies, these discriminatory experiences are strongly associated with negative feelings that significantly jeopardize mental well-being (38, 93, 94).

The second sub-theme was identified through an illness-centered approach, which concerns about manifestations of mental health disruptions, symptoms, and disorders (89). Numerous investigations have delved into mental health from a psychopathological standpoint. The terminologies employed in the included studies encompassed mental ill health, common mental disorders, or psychiatric disorders (39, 42, 52, 64, 69, 71). We opted to label our sub-theme as mental ill health to cover comprehensive evidence concerning mental diagnostics and problematic behaviors. Moreover, the concept of internalizing and externalizing problems was also used. Internalizing problems comprises symptoms of anxiety and depression, i.e., those problems are directed toward oneself (40, 46, 74). Externalizing problems comprise breaking of rules, disruptive, delinquent, ASB, and/or aggressive behavior, mainly toward others (20, 40, 46, 74).

The predominant mental disorders observed in adolescents are depression (43, 51, 53, 57) and anxiety (57, 66, 74). Through this scoping review, we have established an association between social inequality and the prevalence of these mental illnesses among adolescents in Latin America. This association is grounded in the understanding that socioeconomic adversity and discrimination give rise to concerns and stressors that adversely compromise well-being, thereby acting as significant risk factors for the onset of these disorders (11, 12, 95, 96).

The literature reviewed also found an association between social inequality and discrimination with ASB, a heterogeneous concept that encompasses behaviors as diverse as physical fighting, vandalism, stealing, status violation, and disobedience to adults. This association had also been considered by other authors, who reported that ASB in children and adolescents was associated with low SES (97).

Although not among the most prevalent mental health conditions in adolescents, this scoping review identified relevant information concerning the association between PTSD and social inequality experienced by street adolescents (62). PTSD appears when individuals or populations are exposed to highly distressing and traumatic events, such as violence or natural disasters, which jeopardize their physical and emotional well-being (98). Adolescents experiencing social inequality, characterized by highly unfavorable conditions, such as armed conflicts, extreme poverty, or gender-based violence, are at high risk for adverse mental health outcomes and the development of PTSD (98–100).

Finally, the included literature also assessed suicidal behavior among adolescents, which can be associated with psychological, especially affective, disorders (43, 47, 72) and comprises intentional self-harm, suicidal ideation (with or without planning), attempted suicide, and completed suicide (37, 43, 56, 58, 66). During adolescence, the influence of socioeconomic contexts can potentially contribute to inadequate emotional regulation and ineffective coping with stressful situations stemming from social inequality and discrimination. These factors may be associated with deliberate self-harm tendencies (96).

The third sub-theme that emerged from the thematic analysis refers to mental health care for populations facing social inequality. This aspect assumes crucial significance as it highlights how individuals in the most challenging life circumstances often confront scarcity of resources to effectively address their mental health (84, 95). However, this sub-theme addressed the type of interventions that would improve the mental health of adolescents in Latin America and the Caribbean, which respond to the development of strategies that firstly address the underlying causes of social inequality, such as social programs and poverty reduction programs (92, 101). Secondly, it is essential to build community spaces that seek to promote the well-being and quality of life of adolescents, with a focus on primary health care and mental health promotion (102, 103).

## 5 Strengths and limitations

The scoping review approach was convenient in our study because the available scientific literature was extensive. Hence, through this methodology, it was possible to map the information and include various aspects needed to answer the research question and understand how social inequality is associated with mental health. The methodology employed, based on JBI guidelines (28), facilitated the systematic development of each of the six research steps and provided an overview of the available literature on the topic of interest. It is noteworthy that within this methodological framework, risk of bias assessment and critical appraisal are not mandatory, as the primary aim is not to critically synthesize the evidence. Consequently, this scoping review does not comprise critical appraisal. Instead, evaluation of the studies focused on their relevance to the research aim in accordance with the inclusion criteria outlined at the outset. Furthermore, two authors of this study (JCSC and SEAM) are trained by JBI in the development of scoping reviews. Although the main author (JSCS) only has knowledge of Spanish and English, the fact that two authors (SEAM and VAMB) are fluent in Portuguese made it possible to break down the language barrier and broaden the scope of the review. Another strength of the methodology was the involvement of an expert in Information Literacy Education/Systematic Reviews in the construction of the search strategy.

However, it is important to highlight the limitations of this review. Firstly, in formulating the search strategy, terms such as “ethnicity/race”, “migration”, and “gender”, along with closely related terms, were omitted. This decision was made because including these terms redirected the results to issues not within the scope of this review. Consequently, only broader terms such as “social segregation”, “social exclusion”, and “social discrimination”, among others, were included. So most of the available information on intersecting social inequality refers to socioeconomic stratification/class. Thus, it is considered relevant to recognize and expand knowledge about the influence of these three variables related to social inequality and adolescent mental health. While our search strategy includes research from a wide range of countries in the Latin America and the Caribbean (*n* = 42), the final review revealed that the reported research originated from only seven countries (Brazil, Chile, Colombia, Ecuador, Mexico, Nicaragua, and Peru), with none of them considered part of the Caribbean. This highlights the lack of research in the field of social inequality and adolescent’s mental health in the Caribbean region, potentially attributed to limited resources or the stigma surrounding mental health in the area (104, 105). This study underscores the necessity for increased focus on primary research addressing this topic in Caribbean countries. The limited representation of countries may introduce potential bias and restrict the generalizability of our findings to other countries within the region.

The data extraction sheet was designed by one author (JCSC) and discussed among the team (SEAM, VAMB, and KH) before starting the extraction step. The data extraction process was carried out by only one author (JCSC). However, the information extracted from the Portuguese articles was verified by the fluent Portuguese authors (SEAM and VAMB). The thematic analysis was developed by one author (JCSC) and then discussed with three authors (LPG, SEAM, and VAMB) in order to arrive at the refined themes and sub-themes presented in this study. While relying on a single researcher for data extraction and thematic analysis facilitates focused exploration, it may limit diverse insights and overlook data nuances (106). Collaborative analysis involving multiple perspectives might have mitigated these limitations, fostering a more holistic and robust understanding of the data.

## 6 Conclusion

This scoping review represents an overview of the social inequality experienced by adolescents in Latin America and its relationship with mental health outcomes. Scientific literature establishes that social inequality operates at the intersection of ethnicity/race, migration, gender, and socioeconomic class. These dimensions, along with experiences of discrimination and social vulnerability, contribute to the perpetuation of inequality. This review reveals an association between social inequality and adverse mental health outcomes in adolescents. Adolescents exposed to intersecting inequalities reported low levels of well-being as well as higher levels of mental ill health and difficulties accessing mental health care.

In light of these compelling findings, we issue a resounding call to action to academia, government agencies, the private sector, and health systems across the region to develop strategic interventions that address the structural challenges of social inequality. By enhancing living conditions and improving the root causes threatening the well-being and mental health of young people, we can foster a more equitable and healthier adolescence.

## Author contributions

JSCS: Conceptualization, Investigation, Formal Analysis, Writing – original draft, Writing – review & editing. LPG: Conceptualization, Investigation, Writing – original draft, Writing – review & editing. SEAM: Investigation, Writing – original draft, Writing – review & editing. VM-B: Investigation, Writing – review & editing. CS: Conceptualization, Writing – review & editing. KH: Conceptualization, Writing – original draft, Writing – review & editing.
