# Supplementary material for: Mental health among adolescents exposed to social inequality in Latin America and the Caribbean: a scoping review
Source: Front Public Health. 2024 Apr 10;12:1342361. doi: 10.3389/fpubh.2024.1342361 (PMC11041031; doi:10.3389/fpubh.2024.1342361)
Supplement: Supplementary file 1 [file Table_1.docx]

Supplementary Material

Table 1: Characteristics of the included studies

| **Author, year Country (language)** | **Type of article Aim/goal/objective** | **Design/method Time of data assessment Sample size and setting/context Age in years: range (mean) % female gender** | **Assessment tool/method: assessed variables/contents** | **General results** |
| --- | --- | --- | --- | --- |
| Alfaro-Inzunza et al., 2019  Chile (English) | Original article To describe the notions of life satisfaction and dissatisfaction in low socioeconomic status adolescents | Qualitative study/drawing and group performances and semi-structured individual and group  interview Not reported 52 adolescents of low socioeconomic status, coming from different geographical areas  10-14 (not reported) 55.8% | 4 individual interviews: first, participants drew about their feelings towards their family and their neighbourhood; second, interviews focused on explaining and discussing the drawing 8 group interviews: each group was subdivided into pairs or trios, which then demonstrated situations that please and displease them about their school experience. The performance was followed by a discussion | Having harmonious personal relationships, especially with family, teachers, and neighbours, was subjectively important to adolescents and their life satisfaction. When conflicts arise with significant adults such as family break-ups or conflicts between neighbours, life satisfaction diminishes. |
| Alvarado et al., 2001  Chile (Spanish) | Original article To describe inequalities in psychosocial and mental health aspects in young people of different socioeconomic status | Cross-sectional study/survey Not reported  1,250 adolescents from several cities in the Magallanes Region attending 3rd grade in public and private schools  Not reported (not reported) 51.9% | School type, parental educational level and working hours, mental-health-related aspects such as no. of visits to mental health professionals, critical life events, perceived support, self-esteem Goldberg scale (GHQ-30; 30 items): depressive and anxiety symptoms | A strong association was found between school type and the parental educational level as well as the mothers’ working hours outside home. Compared with women at private schools, young women at public schools reported to feel more anxious and/or depressed, less visits to mental health professionals, more critical live events, less perceived support from their parents and friends as well as lower self-esteem. Compared with men at private schools, men at public schools reported less visits to mental health professionals and less support from their friends. |
| Arteche and Ruschel Bandeira, 2003  Brazil (Portuguese) | Original article To assess subjective well-being, comparing adolescents from educational work, regular work and adolescents who do not work | Cross-sectional study/survey Not reported 193 adolescents studying at public schools in the city of Porto Alegre  14-17 (15.7) 42.0% | Socio-Demographic Data Questionnaire (16 items): socioeconomic information  Multidimensional Scale of Child Life Satisfaction adapted for adolescents – (EMSV-A; 61 items): satisfaction with family, self-comparison, friendship, school, nonviolence, self, work | Adolescents who work reported higher scores related to satisfaction with their lives, especially concerning self-comparison. In the comparison between the two groups of young workers, those in educational work were more satisfied with their jobs. |
| Silva et al., 2007  Brazil (Portuguese) | Original article To analyse the factors associated with psychological well-being among adolescents in a southern Brazilian city | Cross-sectional study/survey 2002 960 adolescents from an urban zone in southern Brazil 15-18 (16.5) 51.8% | Socioeconomic status, drug use including smoking and alcohol, and physical activity  Andrews' scale of faces: psychological well-being | Low socioeconomic status and low parental education were associated with lower levels of psychological well-being. Some health behaviours such as sedentary lifestyle, smoking, and alcohol consumption or abuse in the past month were also associated with lower psychological well-being scores. |
| Barcelata Eguiarte et al., 2012  Mexico (Spanish) | Original article To analyse the subjective appraisal of stressful life events in two samples of adolescents of low socioeconomic level in the eastern and suburban areas of Mexico City | Cross-sectional study/survey Not reported 538 adolescents from public middle and high schools located in the State of Mexico 13-18 (15.3) 51.5% 100 adolescent patients in outpatient mental health services of public institutions identified with various emotional and behavioural problems by the health care team 13-18 (14.3) 38.0% | Socioeconomic and demographic variables (33 items)  Life Events Questionnaire for Adolescents (130 items): life events in the last year | Economic restrictions were reported in both the clinical and school samples. However, adolescents in the clinical sample reported greater adversity and worse restrictions such as lack of computer or internet as well as less money per day and low levels of parental education and occupation. Personal and school issues are reported as the most stressful life events in both samples. |
| Souza et al., 2021  Brazil (Portuguese) | Original article To analyse the relationship between families’ social vulnerability with children/adolescents’ well-being | Mixed methods study/survey, body map storytelling, and interviews Not reported  Quantitative stage  37 families (parents and/or guardians) with 48 children/adolescents attending the services for coexistence and strengthening of bonds of the secretary of social assistance of Ribeirão Preto 25-50 (not reported) 89.2%  Qualitative stage  4 children/adolescents from social vulnerable families, 1 coordinator, 3 family members, and 4 teachers of the selected children/adolescents  Not reported (not reported) Not reported | Sociodemographic questionnaire: social vulnerability Body map storytelling and semi-structured interviews: impact of the social vulnerability on health and well-being | Children and adolescents who belonged to the families reporting the highest social vulnerability were going through a process of emotional suffering associated with precarious living conditions. In addition, these families reported lack of employment or occupational opportunities as well as challenges in finding help or receiving necessary support to deal with their problems. It was found that emotional suffering is not easily recognised, so they lack strategies to manage it.  . |
| Benjet et al., 2012  Mexico (English) | Original article To analyse the socio-demographic characteristics and the prevalence of psychiatric disorders, substance use, and suicidal behaviour of adolescents classified as not in education, employment or training (NEET), those who work only, those who both work and study, and those who study only | Cross-sectional study/survey 2005 3005 adolescents from Mexico City 12-17 (not reported) Not reported | World Mental Health adolescent version of the Composite International Diagnostic Interview (WMH-CIDI-A): psychiatric diagnosis, substance use, suicidal behaviour, education, employment and family income  Age, family structure, adult responsibilities | NEET and working adolescents reported lower family incomes and education, family disintegration and having responsibilities such as marriage and children. This is associated with increased risk of psychiatric disorders, substance use, and suicidal behaviour compared to full time students. |
| Bordin et al., 2013  Brazil (English) | Original article To investigate the association between lifetime exposure to exclusively non-harmful paid work activities with emotional and/or behavioural problems in children | Cross-sectional study/survey April 2002 to February 2003 212 adolescents from a typical urban poor neighbourhood of Embu, metropolitan area of São Paulo 9-13 (not reported) 50.0% | Child Behaviour Checklist (CBCL/6–18, 118 items): internalizing and externalizing problems  School delay, grade, school dropout Family economic classification questionnaire by Brazilian Association of Research Companies: family socioeconomic status  Type of adolescent lifetime paid work | Adolescents who have worked are more likely to have internalizing problems than their peers who have never worked. No association was found between educational variables and exposure to work. The socioeconomic status of adolescents who have worked was on average lower than that of their never-worked peers. |
| Butler, 2009  Brazil (English) | Original article To investigate the experiences, identities, and aspirations of street children and adolescents as well as those who have experienced living on the street in Rio de Janeiro, formed as they are around the conditions of exclusion, violence, and discrimination | Qualitative study/life stories interviews  Not reported 92 adolescents/young adults living or who had lived on the streets of Rio de Janeiro, Brazil 11-20+ (not reported) Not reported | Interviews addressing trajectories to and through the streets, reasons and motivations for entering this space, dangers and joys of the street, and to disengage from the street (if it was the case) | Adolescents living on the streets are similar to their peers in aspects such as claiming for citizenship, dreams of family, housing, jobs, and education as well as way of dressing, slang, music, and the aspiration to be treated with respect. However, they differ in the fact that their life on the street, marked by exclusion and unsatisfied needs, leads them to a permanent search for security, respect, care, and freedom. |
| Cucchiaro and Dalgalarrondo, 2007  Brazil (English) | Original article To analyse the association of adolescents’ mental health and quality of life with neighbourhood disadvantage by comparing the two areas of Campinas (central and outer-city) | Cross-sectional study/survey February-October 2002 811 adolescents from two different socioeconomic areas in Campinas, a Brazilian industrialised city 10-16 (13.0) 48.0% | Strengths and Difficulties Questionnaire (SDQ), self-report and teacher versions: prediction of probable mental problems Quality of Life Inventory (ILK): Quality of life in children and adolescents aged ≥ 10 years  Sociodemographic variables including religion  Teacher evaluation: school achievement according | There is no significant difference in the prevalence of mental problems between inner city and outer city areas. Regarding quality of life and academic achievement, male adolescents who reported having no religion, low parental education, and lower socioeconomic status reported the lowest score. |
| Curto et al., 2011  Brazil (English) | Original article To identify environmental factors associated with anti-social behaviour (ASB) among adolescents living in an urban poor community in Brazil | Cross-sectional study/survey 2002 248 adolescents from a typical urban poor neighbourhood of Embu, metropolitan area of São Paulo 11-17 (13.9) 53.2% | Parent-reported variables: Child Behaviour Checklist (CBCL/6–18, 118 items): internalizing and externalizing problems Self-Report Questionnaire (SRQ-20): maternal common mental health disorders Family economic classification questionnaire by Brazilian Association of Research Companies: family socioeconomic status  Mother’s paid work and husband/partner  Self-reported variables: Youth Self Report (YSR/11-18): internalizing problems and externalizing problems | Father's absence was associated with antisocial behaviour. In older adolescents (15-17 years), mother's mental illness was found to be a risk factor for antisocial behaviour. Among adolescents of low socioeconomic status, the mother's paid job is less likely to be associated with antisocial behaviour. |
| da Silva et al., 2010  Brazil (English) | Letter to the editor To report the prevalence of psychiatric disorders in a sample of Brazilian children and adolescents living under conditions of social vulnerability and a history of early emotional stress | Epidemiological report/observation and interviews 2007-2009 351 children and adolescents who are separated from their family because they had run away from home or were sent to foster centres by the justice system 3-19 (not reported) 32.0% | Psychiatric evaluation: children/adolescents were observed alone and in some cases interacting with their parents, legal guardians, or caregivers Semi-structured psychiatric interview: sociodemographic information, medical past and current history, neurodevelopmental history, drug use, physical and sexual abuse, and family-related mental disorders (no information about interviewees in case of young children) | High levels of psychiatric disorders were reported, including substance use, mood and hyperkinetic disorders as well as anxiety disorders related to early emotional distress. Cases of both physical and sexual abuse were also reported. |
| Souza et al., 2008  Brazil (Portuguese) | Original article To measure the prevalence of depressive symptoms in adolescents in Pelotas, Brazil, and identify their associated factors | Cross-sectional study/survey Not reported 1,117 adolescents from urban area of Pelotas 11-15 (13.0) 51.7% | Socioeconomic status, school life, religion, alcohol consumption, conduct disorders  Children’s Depression Inventory (CDI): depressive symptoms | Low socioeconomic status, history of poor academic performance, absence of religious practice, abuse of alcohol in the previous month, and indications of conduct disorders are associated with the presence of high level of depressive symptoms. |
| Doesschate et al., 2012  Ecuador (English) | Original article To investigate the impact of parental migration on psychological wellbeing of left-behind adolescents (LBA) in Ecuador | Cross-sectional study/survey 2011 755 adolescents from Cuenca and Biblian, Ecuador, divided in two groups: left-behind adolescents and not left-behind adolescents  11-16 (13.9) 43.0% | The Strengths and Difficulties Questionnaire (SDQ): emotional and behavioural symptoms  Extract of the Global School-based student Health Survey (GSHS): loneliness, sleeping problems, sadness  Sociodemographic variables: age, gender, migration characteristics, familiar relationships | Adolescents, mainly girls, whose parents were migrants presented behavioural and emotional symptoms. Those adolescents who are able to talk about their problems with others reported fewer emotional problems, as do those who had a good relationship with their parents before they migrated. |
| Etcheverry et al., 2014  Brazil (English) | Original article To investigate the prevalence of depressive symptoms and suicidal ideation in adolescents whose families work as ragpickers in an Brazilian urban area | Cross-sectional study/survey Not reported 239 adolescents whose parents work as ragpickers in Curitiba, Brazil 12-17 (13.4) 54.0% | Children’s Depression Inventory (CDI): depressive symptoms and suicidal ideation | A prevalence of 23% of depressive symptoms and 35% of suicidal ideation was found in ragpickers adolescents, which is higher than the general population’s scores. Girls presented higher prevalence of depressive symptoms than boys did. |
| Fukuda et al., 2012  Brazil (Portuguese) | Original article To investigate the mental health perception of teenagers from different socioeconomic status backgrounds | Qualitative study/Draw-and-Tell Method  Not reported 129 adolescents from a private high school in the Federal District located in an urban area with a high Human Development Index (HDI) 14-17 (16.3) 54.3% 123 adolescents from two public high schools in an urban region with low HDI 14-17 (15.7) 58.8% | Graphic representation about adolescents’ perception of mental health | Adolescents' conceptions of mental health were grounded in both their personal well-being and their social context. Family relationships and interactions were also determinants of the mental health of adolescents in both socioeconomic contexts. |
| Gajaria et al., 2020  Nicaragua (English) | Original article To assess the knowledge, beliefs, and attitudes of teachers about mental illness, its treatment and the needs of students | Qualitative study/grounded theory 2013-2016 43 educators of adolescents aged 13-19 working in schools in León, Nicaragua Not reported (not reported)  Not reported | 5 focus groups: experiences with adolescents’ mental health in Nicaragua | Educators found it challenging to address the mental health needs of young people affected by poverty, domestic violence, or migration. They expressed that it can be very difficult to engage parents in caring for their children's mental health when they live in contexts of social vulnerability. |
| González-Forteza et al., 1998  Mexico (Spanish) | Original article To analyse the interrelationship between daily psychosocial stress, coping responses, and emotional distress in adolescents of low and middle socioeconomic status residing in Coyoacán, south of Mexico City | Cross-sectional study/survey 1995 800 adolescents residents of Coyoacán, Mexico 13-18 (not reported) 52.3% | Psychosocial stress scale (42 items): stressors experienced in the last three months  Coping response scale (30 items): stress coping categories | Adolescents of low socioeconomic status tend to use avoidance strategies to cope with stress and are more susceptible to psychosocial stress. |
| Graham and Pozuelo, 2022  Peru (English) | Original article To explore educational aspirations (and how they compare to occupational and migratory ones) among participants | Panel study/survey Wave 1: May-June 2017  Wave 2*:* February 2020 Wave 1: 400  Wave 2: 301 Youths from San Juan de Lurigancho, a large peri-urban and relatively poor neighbourhood of Lima Wave 1: 18-19 (18.0)  Wave 2: Not reported (21.0) Wave 1: 53.8%  Wave 2: 57.0% | Educational, occupational, and migration aspirations | Most of the adolescents showed aspirations to reach professional and post-graduate levels of education. Half of the respondents remained committed to their aspirations after two years. |
| Guenes de Oliveira and Meirelles Monteiro, 1989  Brazil (Portuguese) | Original article To understand the psychosocial problems and needs of children between 10 and 15 years from low-income families | Qualitative design/ethnographic method and interviews  Not reported 150 adolescents, inhabitants of the "TURURU" community  10-15 (not reported) 52.0% | Script based interviews: socioeconomic-political-cultural context including schooling, familiar dynamics, health/food/hygiene, occupation and working regime, group awareness, self-awareness Ethnographic observation: description of the community from the point of view of its residents; knowledge of their beliefs, values, language (amongst other aspects) | The results show the precariousness of the socioeconomic conditions, lack of affection, premature assignment of responsibilities as well as physical and moral violence resulting in psychosocial deprivation and vulnerability to mental disorder. |
| Hallal et al., 2010  Brazil (English) | Original article  To assess the well-being in adolescents from a birth cohort in Pelotas, southern Brazil | Cohort study/survey 2004-2005 4,452 adolescents, complete cohort born in 1993 in the city of Pelotas 11 (11) Not reported | Graphic scale of seven faces: well-being.  Demographic and socioeconomic variables | In general, adolescents who are girls, black, or brown reported higher levels of well-being than boys and white people, respectively. Adolescents of low socioeconomic status reported the highest percentage of moderate and low being. |
| Harpham et al., 2004  Colombia (English) | Original article To analyse the association between social capital and mental health, when taking into account an array of demographic variables | Cross-sectional study/survey 2002 1,168 adolescents from Aguablanca, eastern fringe of the city of Cali 15-25 (not reported) 58.0% | Self-Reporting Questionnaire (SRQ-20, 20 items): non-specific psychological distress  Individual’s perceptions and experience of social capital  Basic demographic data: age, sex, length of residence in the neighbourhood, occupation, schooling, household structure, quality of housing, and degree of overcrowding. | The most significant risk factors for psychological distress are being female, having limited schooling, working in the informal sector and having low trust in people. |
| Harpham et al., 2005  Colombia (English) | Original article  To investigate the associations of common mental disorders among low income young people in the city of Cali, Colombia, with violence and social capital | Mixed methods study/survey and focus group  2002; 2004  1,168 adolescents from Aguablanca, eastern fringe of Cali 15-25 (not reported) 58.0% | Quantitative instruments  Self-Reporting Questionnaire (SRQ-20, 20 items): non-specific psychological distress  Adapted ACTIVA violence questionnaire: norms and consequences regarding violence inside and outside home  Basic demographic data: age, sex, schooling Focus group: participants views of social capital and violence in the family and neighbourhood | The most common risk factors for developing psychological distress are being female, having a low level of education and living with violence. The focus groups showed that poverty is the cause of many tensions both within the family and in public life. |
| Herrera et al., 2006  Nicaragua (English) | Original article To identify triggers and processes leading to suicidal behaviour among female adolescents in León, Nicaragua | Qualitative study/grounded theory and content analysis 2001 8 female adolescents admitted to the Heodra hospital, in Leon, Nicaragua, after a suicide attempt 12-19 (15.9) 100% | Individual in-depth interviews: family functioning, childhood/teenage period, friendships, romantic relationships, life projects, and suicidal behaviour (suicide ideation and suicide attempts) | A tentative model of the pathways leading to suicidal behaviour in female adolescents was described. It was found that structural conditions such as dysfunctional families, absent parents and lack of integration induce adolescent girls to emotional distress. Faced with this, they take different actions to solve their problems, but when they fail, they attempt suicide. |
| Hinostroza-Gastelú et al., 2011  Peru (Spanish) | Original article To investigate the association of family dynamics with manifestations of depression in schoolchildren | Cross-sectional study/survey Not reported 152 adolescents from a school in Puente Piedra, Lima 13-15 (13.7) 51.3% | Children’s Depression Inventory (CDI): Depressive symptoms and suicidal ideation Familiar APGAR: family functionality according to adaptability, emotional climate, affectivity, communication, and development | The greatest number of depressed adolescents were found in families with good family functionality. Within the population, there were manifestations of pessimism and social isolation as well as poor school performance and decreased concentration. Most of the depressed youths are affected by poverty. |
| Lemos Couto et al., 2020  Brazil (English) | Original article To analyse the emotional/behavioural problems of adolescents showing high social vulnerability from parents’ or guardians’ point of view | Cross-sectional study/survey Not reported 274 parents or guardians responsible for adolescents from municipal education system schools in Salvador, Brazil  11-17 (13.3)  46.7% (female adolescents) | Sociodemographic Questionnaire (QSD, 10 items): sociodemographic information  Child Behaviour Checklist (CBCL/6–18, 118 items): internalizing and externalizing problems | From the perspective of parents of socially vulnerable adolescents, the majority of youths experienced somatic complaints, followed by anxiety and depression (internalizing). Additionally, aggressive behaviour was frequently reported (externalizing). |
| Lucio et al., 2001  Mexico (Spanish) | Original article To describe inequalities in psychosocial and mental health aspects in young people of different socioeconomic status | Cross-sectional study/survey Not reported 1,071 adolescents from Distrito Federal, Mexico 13-18 (not reported) 51.1% | Life Events Questionnaire (71 items): stressors experienced related to family, school, social life, sexuality, achievements and failures, economic resources, health, and behavioural problems | Adolescents with a low socioeconomic status reported more subjectively negative stressful events compared with adolescents with a high socioeconomic status. Older adolescents reported more stressful life events. |
| Palomar-Lever and Victorio-Estrada, 2016  Mexico (English) | Original article  To identify the predictive factors for the internalizing and externalizing problems in adolescents registered for a Mexican government programme | Cross-sectional study/survey Not reported  540 adolescents registered for a Mexican government programme against poverty  12-19 (14.9) 43.7% | Youth Self Report (YSR/11-18): internalizing externalizing problems  Stressful events, resilience, direct-reflexive coping, authoritarian parenting style  Gender and Type of Locality | Females report higher levels of internalizing problems, while males reported higher levels of externalizing problems. Higher scores of authoritarian parenting style, frequency of stressful events, lower level of resilience, and direct coping style as well as living in a rural area are associated with internalizing and externalizing problems. |
| Pluck et al., 2015  Ecuador (English) | Original article  To investigate the prevalence of post-traumatic stress disorder (PTSD) in Ecuadorian street adolescents | Cross-sectional study/survey Not reported 37 street adolescents recruited from a charitable social project from Quito, Ecuador 10-17 (13.5) 24.3% | Events particularly frightening, violent, or dangerous  UCLA PTSD Index for DSM IV-Adolescent Spanish version (49 items): post-traumatic stress disorder | Three quarters of the adolescents (75.7%) reported incidents in which they were exposed to interpersonal violence. A total of 59.5% met the criteria for post-traumatic stress disorder. |
| Quintero-Jurado and Ossa-Henao, 2018  Colombia (Spanish) | Original article To investigate youth-focused groups' roles in promoting mental health and preventing mental health issues among young people | Qualitative study/hermeneutic method Not reported 6 community groups aimed at young people led by community members in the municipality of Bello, Antioquia 14-24 (not reported) Not reported | 6 focus groups with community groups’ members and 5 semi-structured interviews with each community group leader: expectations, results, achievements, and benefits of participation in the community group for mental health | Although created for learning a skill such as music or sport, the community groups became safe spaces for the adolescents to spend their leisure time as well as to establish social relationships and life plans. Thus, these groups became a setting for mental health promotion. |
| Raffaelli et al., 2007  Brazil (English) | Original article To assess the exposure to different developmental risks and associations between risk factors and psychosocial well-being | Cross-sectional study/survey 2000 918 adolescents/young adults in the city of Porto Alegre, Brazil, in schools in impoverished neighbourhoods and in institutions serving out-of-school youth, e.g., nongovernmental organizations, community centres 14-19 (15.8) 51.9% | Sociodemographic variables, gender, community risk factors, economic risk factors, family risk factors, psychological adjustment, behavioural adjustment | Adolescents in the poorest areas of Brazil exposed to higher risks related to living in dangerous communities, poverty, and family difficulties reported lower levels of psychological and behavioural adjustment. Gender analysis found that males experienced higher risks associated with community, and females were more exposed to risks related to family and economic conditions. |
| Romo et al., 2016  Ecuador (English) | Original article To analyse the association of hunger, parental/guardian involvement, and experiences of peer victimization with symptoms of depression and suicidal ideation among Ecuadorian adolescents | Cross-sectional study/survey 2007 5,524 adolescents at schools in Quito, Guayaquil, and Zamora, Ecuador  10-17 (not reported) 50.4% | Subset of the measures assessed in the Global School-based Student Health Survey 2007 (GSHS): depressive symptoms and suicidal ideation, adolescent hunger, parental/guardian involvement, peer victimization | High levels of hunger (prevalence: 41.2%), low levels of parental involvement, and peer victimization found among adolescents were associated with depressive symptoms and suicidal ideation. |
| Saad et al., 2004  Ecuador (Spanish) | Original article To investigate the psychological consequences among remaining adolescent family members after emigration of at least one family member | Cross-sectional study/review of medical records  2002 61 medical records of patients attended for the first time at the Adolescent Unit of the "Lorenzo Ponce" Psychiatric Hospital in Guayaquil, who had at least one family member who had emigrated 11-18 (14.2) 49.2% | Data extraction matrix (16 items): socio-demographic characteristics, family members who migrated, conditions of migration, family structure, mental ill health status/diagnosis | Depressive disorders accounted for most of the pathologies found in adolescents who were relatives of migrants. In all cases, the symptomatology appeared after the adolescent's family had migrated. Only 11.5% reported a family history that could be associated with the development of the illness. In most cases, one of the parents migrated, resulting in the need to modify the family structure. |
| Santana et al., 2007  Brazil (English) | Original article To analyse the association of having black skin colour and perceived experience of racial discrimination with depressive symptoms among adolescents, taking into consideration age, gender, and socioeconomic status | Cross-sectional study/survey 2000 973 adolescents living in Salvador, capital city of Bahia State, Brazil  10-21 (not reported) 68.9% | Skin colour, perceived racial discrimination, suicidal behaviour, feeling unhappy or unhealthy, self-esteem. Patient Health Questionnaire (PHQ) self-report version: depressive symptoms | People from the black population or who reported some form of racial discrimination were less likely to show low levels of self-esteem. However, feelings of unhappiness or being unhealthy, higher prevalence of depressive symptoms as well as high prevalence of suicidal behaviour was found in those who reported perceived racial discrimination. |
| Scivoletto et al., 2011  Brazil (English) | Intervention development article  To describe the implementation, operation and challenges of The Equilibrium Project (TEP) through a community-academic partnership | Implementation of a project to integrate diverse services for street children/adolescents 2007-2009 351 adolescents served in TEP: street children/adolescents, other children in need of psychiatric care  Not reported (12.5) 32.0% | TEP provides initial psychiatric assessment, development of an individualised care plan, and long-term case monitoring. TEP promotes the development of communication skills that generate opportunities for safe socialization. | Young people served in TEP present emotional stress, such as neglect or physical abuse, and a high prevalence of psychiatric disorders, so the caring staff must specialise in these fields. After two years of operation, more than half of the participants had completed or were continuing the programme. |
| Serrano-Ruiz and Olave-Chaves, 2017  Colombia (Spanish) | Review article To identify the main personal, family, and social factors associated with suicidal behaviour in adolescents | Literature review 2016 49 subject reviews, original articles, or systematic reviews, institutional pages of governmental entities, and books written in Spanish or English 11-19 (not reported)  Not reported | Data extraction sheet: personal, family, educational, and socioeconomic factors associated with suicidal behaviour in adolescents | Low socioeconomic status, low educational level, and unemployment were identified as risk factors for suicidal behaviour in adolescents. It was also reported that conflicts within the family, often connected to economic problems, were associated with stress and suicidal behaviour among adolescents. |
| Sherman et al., 2011  Brazil (English) | Original article To analyse risk and protective factors associated with adolescent mental health problems in the context of social and economic disparity in Bahia, Brazil | Cross-sectional study/survey Not reported 344 adolescents from a public school in a municipality outside of Salvador, Bahia 11-18 (13.6) 61.0% | Youth Self Report (YSR/11-18): internalizing externalizing problems  Sociodemographic variables: age, gender, race, parents’ marital status, parents’ education. Family Environment Scale: family conflict and family cohesion | Female adolescents with unmarried or low-educated parents reported greater risk of reporting internalizing and externalizing problems. Family conflicts are related to internalizing and externalizing problems in both girls and boys. Likewise, family cohesion decreases the risk of the presence of these problems. |
| Sladek et al., 2020  Colombia (English) | Original article To investigate associations among ethnic-racial discrimination, ethnic-racial identity (ERI), and psychosocial adjustment (i.e., self-esteem and depressive symptoms) among Colombian adolescents | Cross-sectional study/survey 2017-2018 462 adolescents in high school that reflect socioeconomic diversity in Medellin, Colombia  Not reported (15.9) 47.3% | Rosenberg Self-Esteem Scale (10 items): self-esteem Centre for Epidemiologic Studies Depression Scale (20 items): depressive symptoms  Adolescent Discrimination Distress Index: ethnic-racial discrimination Ethnic Identity Scale—Brief: ethnic-racial identity | Almost 40% of the adolescents reported having been victims of racial/ethnic discrimination in the last year. This was associated with low self-esteem and depressive symptoms. Adolescents who felt comfortable with their racial identity reported better self-esteem scores and fewer depressive symptoms. |
| Varela et al., 2020  Chile (English) | Original article To investigate the effect of bullying on adolescents’ subjective well-being while considering the levels of socioeconomic status of the schools where bullying occurs | Cross-sectional study/survey 2018 1,914 adolescents from 26 urban schools in Chile. Not reported (11.6) 47.1% | Students' Life Satisfaction Scale (SLSS): students’ self-satisfaction Brief Multidimensional students' life satisfaction scale (BMSLSS): subjective perceptions about well-being, namely family, friends, school, and life in general  Overall Life Satisfaction (OLS) scale: whole life satisfaction Illinois Bullying Scale (IBS): forms of bullying behaviour: being bully, fighting, and victim School Vulnerability Index (IVE in Spanish): students’ socioeconomic status | Students with lower SES tend to report lower levels of life satisfaction compared to those with higher SES. Moreover, as SES decreases, the likelihood of being a victim of bullying tends to increase. |
| Viñas et al., 2019  Brazil (English) | Original article To analyse the relationship between subjective social well-being and coping strategies to mediate the experience with stress | Cross-sectional study/survey Not reported 864 adolescents in the final year of primary and first year of secondary school in the province of Ceará, Brazil 10-15 (11.9) 47.8% | Multidimensional Poverty Index (MPI) Overall Life Satisfaction (OLS) scale: whole life satisfaction  Personal Wellbeing Index – School Children (PWI-SC): leisure time and experience at school satisfaction Students' Life Satisfaction Scale (SLSS): students’ self-satisfaction  The Schoolagers' Coping Strategies Inventory (SCSI): coping strategies | The higher the MPI, the lower were the life satisfaction scores. The dissatisfaction increases with age. Coping and distraction strategies seem to be relevant for improving life satisfaction. |
| Webb and Alvarez, 2018  Chile (English) | Original article To investigate the role of friendship dynamics among newcomer Latin American migrant youth during their transitions to Chilean high schools | Qualitative study/case study  Not reported 10 adolescents from two different schools in Santiago, Chile, most from lower-middle or low-income families 14-18 (15.5) 40.0% | Observation: friendship dynamics and activities in recreational spaces  Life history interviews: experiences of education in the countries of origin, transitions to the Chilean school, friendships, and experiences of bullying or victimization | Although most of the participants reported having been victims of ethnic discrimination, they were able to manage it through inter-ethnic friendships, which they were able to build through different strategies of inclusion in local groups. |
| Zimmerman et al., 2022  Colombia, Mexico, and South Africa (only data from Colombia and Mexico were considered in this research) (English) | Original article To investigate the relationship between multidimensional poverty and youth depressive symptoms | Cross-sectional study/survey Colombia  2015  3,607 young participants in a national survey 11-25 (18.0) 56.0%  Mexico 7,405 young people from 8,400 households from Mexico 11–25 (20.0) 55.0% | Colombia:  Self-Reporting Questionnaire (SRQ-20, 13 items): depressive symptoms  Mexico Clinical Questionnaire for the Diagnosis of Depressive Syndrome (CCDSD in Spanish, 21 items): depressive symptoms  All countries: Colombian Multidimensional Poverty Index (CMPI): health and access to public utilities, housing conditions | In Colombia and Mexico, a significant association was found between multidimensional poverty and depressive symptoms. Only in Colombia, young people with poor access to health services and prolonged unemployment reported higher scores of depressive symptoms. |
