# Supplementary material for: Mental health among adolescents exposed to social inequality in Latin America and the Caribbean: a scoping review
Source: Front Public Health. 2024 Apr 10;12:1342361. doi: 10.3389/fpubh.2024.1342361 (PMC11041031; doi:10.3389/fpubh.2024.1342361)
Supplement: Supplementary file 2 [file Data_Sheet_1.docx]

Supplementary Material

# Appendix 1: Search strategy used in Medline via OVID

Adolescent/ OR Adolesc*.ab,ti. OR Adolescent/ OR Teen*.ab,ti. OR Youth.ab,ti.

**AND**

Latin America/ OR OR exp Caribbean Region/ OR exp Central America/ OR exp South America/ OR Mexico/ OR Latin America.ab,ti. OR Caribbean.ab,ti. OR Central America.ab,ti. OR South America.ab,ti. OR (Antigua and Barbuda).ab,ti. OR Aruba.ab,ti. OR Bahamas.ab,ti. OR Barbados.ab,ti. OR Cuba.ab,ti. OR Dominica.ab,ti. OR Dominican Republic.ab,ti. OR Grenada.ab,ti. OR Guadeloupe.ab,ti. OR Haiti.ab,ti. OR (Cayman Islands).ab,ti. OR (Turks and Caicos Islands).ab,ti. OR (Virgin Islands).ab,ti. OR Jamaica.ab,ti. OR Martinique.ab,ti. OR (Puerto Rico).ab,ti. OR (Saint Barthelemy).ab,ti. OR (Saint Kitts and Nevis).ab,ti. OR (Saint Vincent and Grenadines).ab,ti. OR (Saint Lucia).ab,ti. OR (Trinidad and Tobago).ab,ti. OR Belize.ab,ti. OR (Costa Rica).ab,ti. OR (El Salvador).ab,ti. OR Guatemala.ab,ti. OR Honduras.ab,ti. OR Nicaragua.ab,ti. OR Panama.ab,ti. OR Argentina.ab,ti. OR Bolivia.ab,ti. OR Brazil.ab,ti. OR Chile.ab,ti. OR Colombia.ab,ti. OR Ecuador.ab,ti. OR Guyana.ab,ti. OR (French Guyana).ab,ti. OR Paraguay.ab,ti. OR Peru.ab,ti. OR Suriname.ab,ti. OR Uruguay.ab,ti. OR Venezuela.ab,ti. OR Mexico.ab,ti. OR Latin*.ab,ti.

**AND**

Social Deprivation/ OR social vulnerability/ OR Social Conditions/ OR Poverty/ OR Social Marginalization/ OR Social Segregation/ OR Social Class/ OR Social Discrimination/ OR social inequality.ab,ti. OR social inequity.ab,ti. OR Social disadvantage.ab,ti. OR social segragation.ab,ti. OR Social exclusion.ab,ti. OR Social deprivation.ab,ti. OR Social Vulnerability.ab,ti.OR Social Marginalization.ab,ti. OR social gaps.ab,ti. OR social defeat.ab,ti. OR social class.ab,ti. OR socioeconomic.ab,ti. OR income.ab,ti. OR social discrimination.ab,ti.

**AND**

Mental Health/ OR Self Efficacy/ OR Personal Autonomy/ OR Emotions/ OR Affect/ OR

Psychology, Adolescent/ OR Mental health.ab,ti. OR Well-being.ab,ti. OR Wellbeing.ab,ti.

Self-efficacy.ab,ti. OR Autonomy.ab,ti. OR Feel*.ab,ti. OR Emotion*.ab,ti. OR enjoy.ab,ti.
